# D614G reshapes allosteric networks and opening mechanisms of SARS-CoV-2 spikes

**DOI:** 10.1073/pnas.2504793123

**Published:** 2026-05-08

**Authors:** Fiona L. Kearns, Anthony T. Bogetti, Carla Calvó-Tusell, Mac Kevin E. Braza, Lorenzo Casalino, Amanda J. Gramm, Sean Braet, Mia A. Rosenfeld, Harinda Rajapaksha, Bryan Barker, Ganesh Anand, Lillian T. Chong, Surl-Hee Ahn, Rommie E. Amaro

**Affiliations:** ^a^Department of Molecular Biology, University of California San Diego, La Jolla, CA 92093-0340; ^b^Department of Chemistry, University of Pittsburgh, Pittsburgh, PA 15260; ^c^Department of Chemistry and Biochemistry, University of California San Diego, La Jolla, CA 92093-0340; ^d^Department of Chemistry, Pennsylvania State University, University Park, PA 16802; ^e^Oracle for Research, Oracle Cloud, Austin, TX 78741; ^f^Department of Chemical Engineering, University of California Davis, Davis, CA 95616

**Keywords:** SARS-CoV-2 spike glycoprotein, COVID19, weighted ensemble simulations, receptor binding domain opening, ACE2 binding

## Abstract

The severe acute respiratory syndrome coronavirus 2 spike glycoprotein enables infection through a key conformational transition that exposes its receptor binding domain (RBD). Experimental evidence indicates that spike mutations, particularly the early D614G variant, alter the rate of this conformational shift, potentially increasing viral infectivity. We conducted extensive weighted ensemble simulations of the Ancestral, Delta, and Omicron BA.1 spike strains to investigate relationships between sequence mutations and RBD opening dynamics. We observe that Ancestral, Delta, and Omicron BA.1 spike RBDs open differently. Via dynamical network analysis, we identified two allosteric communication networks connecting all S1 domains: the established N2R linker and a newly investigated antiparallel R2N linker. In Delta and Omicron BA.1 variant spikes, RBD opening is facilitated by both linkers, while the Ancestral strain relies predominantly on the N2R linker. In the Ancestral spike, the D614-K854 salt bridge impedes allosteric communication through the R2N linker, whereas the loss of this salt bridge in all subsequent variants of concerns allows for increased local flexibility, thereby accelerating RBD opening. Hydrogen–deuterium mass spectrometry experiments validate these altered dynamics in the D614 region. This study unveils a “hidden” network, connecting the N-terminal domain to the RBD via the 614-proximal region, and the D614G mutation reshapes the fitness landscape of these critical viral glycoproteins.

## Significance

Our work reveals how the D614G mutation in the severe acute respiratory syndrome coronavirus 2 (SARS-CoV-2) spike protein reshapes its internal communication pathways and speeds up receptor binding domain (RBD) opening, providing mechanistic insight into the enhanced infectivity of SARS-CoV-2 variants of concern. We also describe differences in opening pathways for Delta and Omicron BA.1 spike RBDs relative to the original (Ancestral) coronavirus strain from Wuhan, China.

**Fig. 1.**
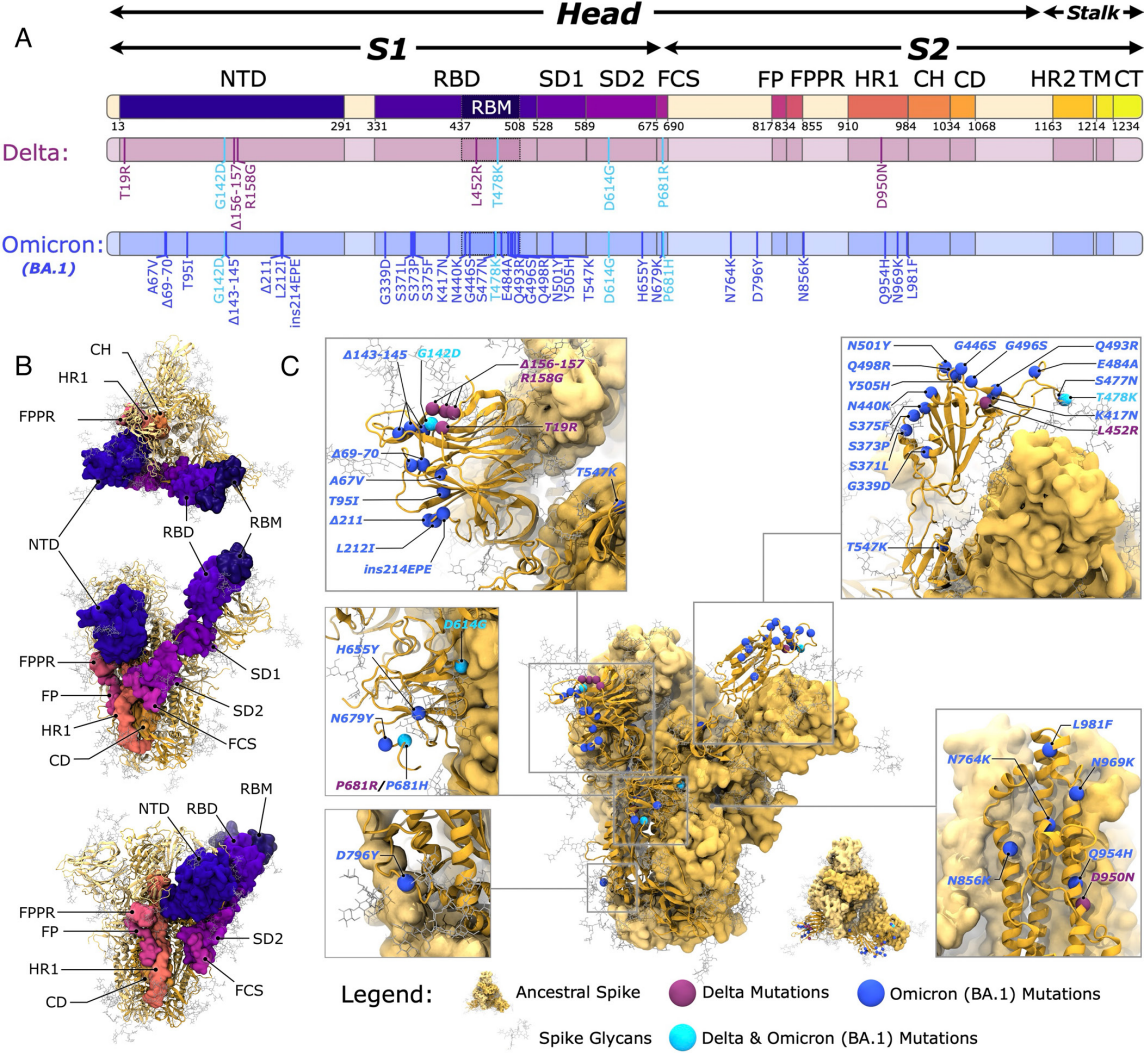
Mutations and domain organization of the Ancestral, Delta, and Omicron BA.1 spike proteins. (*A*) Spike domain definitions and mutations along the 1D sequence. Domains: head (13 to 1140), stalk (1141 to 1273), S1 (13 to 685), S2 (686 to 1140), NTD (13 to 291), RBD (331 to 528), RBM (437 to 508), SD1 (529 to 589), SD2 (590 to 675), FCS (675 to 690), FP (817 to 834), FPPR (835 to 855), HR1 (910 to 984), CH (985 to 1034), CD (1035 to 1068), HR2 (1163 to 1210), TM (1214 to 1234), and CT (1235 to 1273). (*B*) *Top* and side views of the 1-RBD-up spike head highlighting domains from (*A*). Chain A is shown as a space-filling surface; chains B and C as yellow ribbons. Glycans are shown as light-gray licorice. (*C*) Positions of Delta and Omicron BA.1 mutations on the spike head. The Ancestral spike is shown as yellow (chain A ribbon; chains B, C surface). Glycans are light gray. Delta-specific, Omicron-specific, and shared mutations are shown as purple, blue, and cyan beads, respectively.

The SARS-CoV-2 (Severe Acute Respiratory Syndrome Coronavirus 2) replication cycle in humans begins with host-cell binding and membrane fusion (1), both mediated by the homotrimeric spike (S) glycoprotein (1). During expression and viral assembly, spike folds into a metastable prefusion conformation, with a head region supported by a membrane-anchored stalk (Fig. 1*A*). To prime the spike for its class I fusion role, two key events occur. First, furin cleaves the RRAR furin cleavage site (FCS), splitting the spike into S1 and S2 domains (Fig. 1 *A* and *B*) (2). Second, in this metastable state, the ACE2 binding receptor binding motif (RBM) within the receptor binding domain (RBD) is shielded by neighboring protein surfaces and N- and O-linked glycans (3). The “closed” prefusion spike must therefore transition to an “open” state in which one or more RBDs shift upward (1-up, 2-up, 3-up states) to expose the RBM (Fig. 1*B*) (1–3). RBD opening also triggers movements in the N-terminal domain (NTD), subdomain 1 (SD1), subdomain 2 (SD2), and the fusion peptide proximal region (FPPR) (Fig. 1 *A* and *B*) (1–4).

Numerous monoclonal antibodies target key epitopes within the RBD and RBM, making spike the basis for all SARS-CoV-2 vaccines, which employ modified spike mRNA sequences or attenuated viruses displaying spike glycoproteins (5–7). Shielding RBD and RBM epitopes until host cell approach may balance infectivity and immune evasion (8). RBD binding can also affect ACE2 enzyme activity (9–11). Following ACE2 engagement, the S1 domain dissociates, exposing the S2 core and fusion peptide (FP) (Fig. 1 *A* and *B*) (4, 12). After all S1 domains are released and FP is freed by a second cleavage (12, 13), the spike undergoes a dramatic transition to the postfusion state, driving host-cell membrane penetration (4, 14). Given its central role in infection and as a proven vaccine antigen, spike structural biology remains a major focus for antiviral drug design (15, 16) and pancoronavirus vaccine development (17–20).

Understanding spike biophysics reveals adaptive pressures driving SARS-CoV-2 evolution (21, 22). Following the initial sequencing of the SARS-CoV-2 genome in late 2019, a new clade (G) emerged in February 2020, characterized by four mutations including a key substitution of aspartic acid to glycine at position 614 of spike (23). By March 2020, this “D614G variant” became the dominant global strain (23). Although residue 614 lies over 100 residues and ~75 Å from the RBM center in the closed state, epidemiological data showed D614G viruses were 4-9× more infectious than the original 2019 (“Ancestral”) strain (23). The variant also correlated with higher viral titers (23–25), higher S incorporation on the virion (26–28), and decreased premature S1-shedding (26). Structural studies sought to explain these effects. While reports differ on whether the D614G mutation increases (29, 30), decreases (25), or leaves ACE2 affinity unchanged (26, 27), most agree it raises the population of open (1-up, 2-up, or 3-up) RBD states relative to the Ancestral spike (25, 28, 31, 32). Cryo-EM structures revealed that D614G disrupts a hydrogen bond to T859 (29) or a salt bridge to K854 (28, 29) on the adjacent S2 domain within the Fusion Peptide Proximal Region (FPPR, Fig. 1 *A* and *B*). The loss of these interactions may increase local flexibility, stabilizing the trimer and reducing S1 shedding (26). Zhang and Cai et al. found that D614G also induces slight outward motion of the SD1 domain, allowing the “630-loop” to pack more tightly toward the core (28). Molecular simulations by Dokainish and Sugita showed this loop is disordered in the Ancestral spike, but becomes ordered and stabilized by the D614G substitution (33). Dokainish and Sugita (33) and Yang et al. (31) further proposed that protonation of D614 at low pH could break the K854 salt bridge, influencing spike conformational states. Despite extensive structural and computational investigations, the allosteric link between D614G and increased RBD openness—and thus increased ACE2 binding, cell fusion, and infectivity—remains unresolved.

By mid-2020, the D614G variant dominated global SARS-CoV-2 circulation (23, 34). Its increased infectivity and faster replication accelerated genomic diversification, leading to many new variants of concern (VOCs), all retaining the D614G mutation (34). Two major examples are Delta and Omicron BA.1 (hereafter “Omicron”), first detected in October 2020 and November 2021, respectively (22, 35). The Delta spike contains 9 mutations relative to the Ancestral sequence, Fig. 1, including T19R, which removes the N-linked glycan at position 17 (36). Early structural studies showed refolding of multiple loops in the NTD and repositioning of the N149 glycan (36–38) likely reducing immune recognition (36). While no large scale rearrangements occur in Delta RBDs or RBMs, the L452R and T478K mutations likely enhance ACE2 binding (39, 40), and P681R and D950N are predicted to increase furin cleavage efficiency and membrane fusion, respectively (41, 42). Omicron’s spike, by contrast, carries 34 mutations relative to the Ancestral strain, Fig. 1, including three deletions and one insertion that initially confounded sequencing and challenged tracking efforts (43–45). Once identified, Omicron rapidly surpassed all earlier variants in transmissibility and hospitalizations (44–46). Its high infectivity is attributed to extensive sequence changes—particularly 15 mutations in the RBD (10 within the RBM) and 8 in the NTD—that promote immune evasion, while strengthening ACE2 binding (47, 48). Cryo-EM and epitope mapping further show that Omicron RBDs favor the up/open conformation relative to Ancestral spikes (49).

Molecular dynamics (MD) simulations have been invaluable during the SARS-CoV-2 pandemic, particularly for revealing structure–function–dynamics relationships in the spike glycoprotein and its otherwise “invisible” glycans (50–52). Using conventional MD and biolayer interferometry (BLI), we showed that glycans at positions N165 and N234 act like “kickstands,” stabilizing RBDs in the up conformation (50). Capturing such large-scale transitions, however, is often intractable without enhanced sampling techniques due to the long timescales involved (53). To overcome this, we used weighted ensemble (WE) enhanced MD to efficiently simulate RBD opening (52). These simulations identified the N343 glycan as a wedge that stabilizes the open RBD through a hydrophobic stacking network (52). BLI confirmed that removing this glycan decreased ACE2 binding, likely by decreasing the population of up RBDs and exposed RBMs (52). From these WE trajectories, we also characterized key salt-bridge and hydrogen-bond interactions that form and break during RBD opening, highlighting residues where mutations could alter dynamics (52). In related work, we have used WE MD to model S2-domain splaying, providing insights that guided stabilizing mutations for pancoronavirus vaccine design (20). Similarly, Lynch et al. have recently used WE MD to reveal how ligand binding modulates Hepatitis B viral capsid assembly (54).

The WE method, first introduced by Huber and Kim for Brownian dynamics (55), enhances sampling of rare barrier-crossing events by running multiple trajectories and periodically resampling based on a progress coordinate (55, 56). Trajectories that advance are replicated, while less-progressed ones are pruned; each carries a statistical weight that maintains unbiased dynamics (55, 56). This conceptually simple and rigorous framework has found applications beyond molecular simulations, including systems biology, meteorology, and astronomy (53). Recent developments, particularly in the open-source WESTPA platform, have extended WE to large molecular systems such as viral glycoproteins.

Here, we used WE simulations with a minimal adaptive binning scheme (57) to efficiently model RBD opening in the Ancestral, Delta, and Omicron spike glycoproteins. Our simulations reveal that the D614 to K854 salt bridge must break before RBD opening in the Ancestral strain and reveal an allosteric network transmitting signals from the NTD to the opening RBD within the same chain—a network strengthened by the D614G mutation. We also examine biophysical factors such as 630-loop flexibility and RBD motion differences among the Ancestral, Delta, and Omicron spikes.

## Results and Discussion

**Fig. 2.**
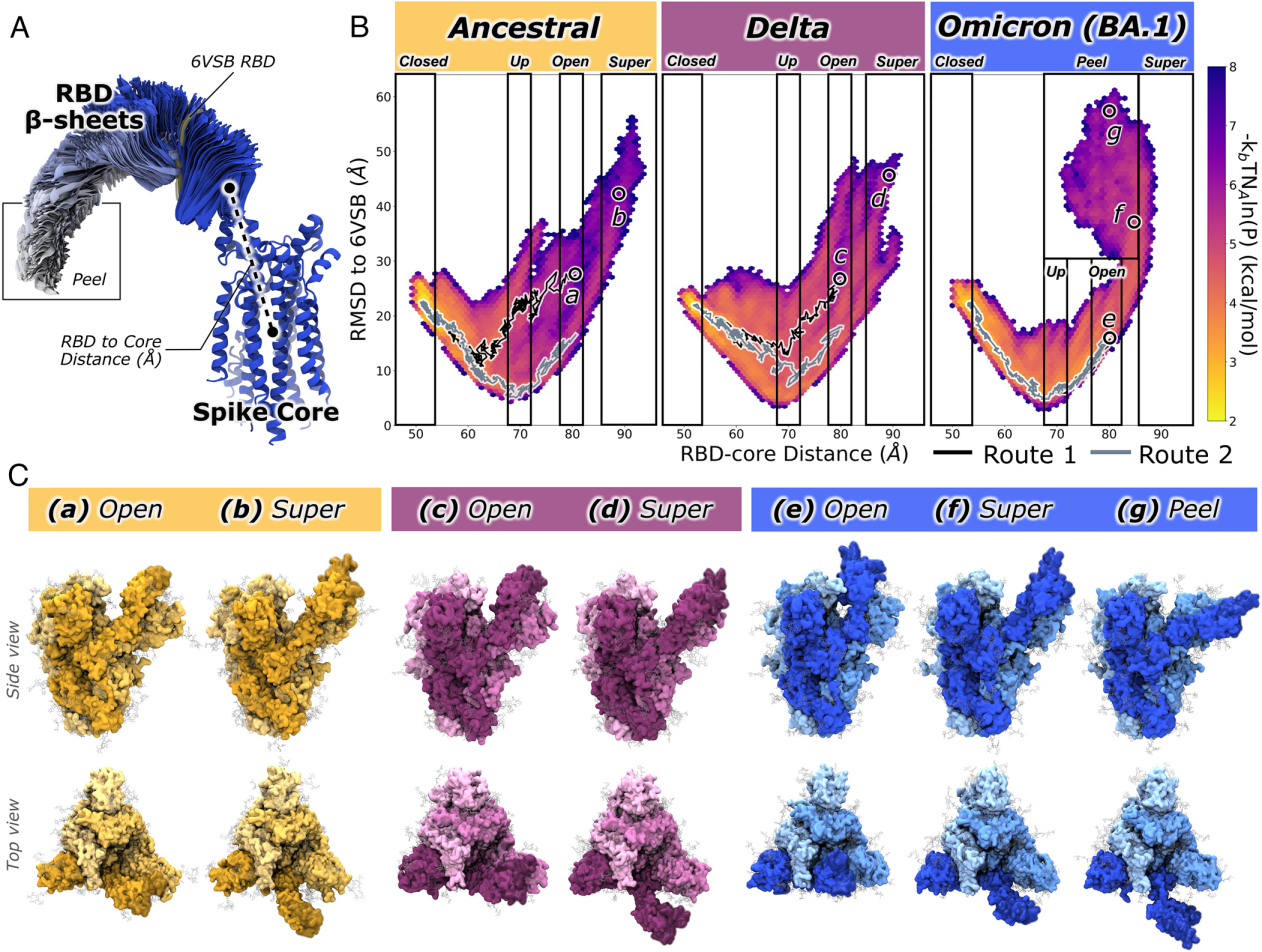
Conformational landscapes for Ancestral, Delta, and Omicron BA.1 spike RBD openings. (*A*) Two-dimensional progress coordinates used in WE simulations to promote RBD opening. Omicron trajectories illustrate the peel conformation. The spike core (α-helical bundle; residues 747 to 785, 945 to 1035) is shown as dark (chain A) and light (chains B, C) blue ribbons. RBD β-sheets (residues 353 to 358, 375 to 381, 394 to 403, 430 to 438, 507 to 516) are colored by state: closed to up (dark blue), open (light blue), and super-open/Peel (white). The 6VSB RBD β-sheets, used for RMSD reference, are indicated. (*B*) Free-energy landscapes (kcal/mol) showing RBD conformational probabilities along the 2D progress coordinate for each variant. Closed, up, open, super-open, and peel regions are labeled; representative routes 1 (black) and 2 (gray) are overlaid. (*C*) Representative open, super-open, and peel conformations for Ancestral (*a* and *b*), Delta (*c* and *d*), and Omicron BA.1 (*e*–*g*) spikes, with their positions mapped onto panel *B*.

We performed equilibrium WE simulations of the Ancestral, Delta, and Omicron spike proteins following Sztain et al. (52). RBD opening was defined using a two-dimensional progress coordinate: 1) the distance between the centers of mass of the RBD β-sheet C_α_ atoms and the spike’s central helix C_α_ atoms, referred to as the RBD-core distance (Fig. 2*A*), and 2) the RMSD between RBD β-sheet C_α_ atoms in each frame and those in the open-state reference structure PDB ID 6VSB (3). This prefusion stabilized spike, which served as the foundation for early mRNA vaccines, provides an ideal reference for RBD opening (3).

While WE sampling proceeds along predefined coordinates and may undersample events outside that space, our focus here is on comparing RBD opening dynamics among variants to reveal major structural differences. Notably, unique conformational states still emerged that were not specified a priori. All simulations included N- and O-linked glycans modeled as in Casalino et al. (50) and Watanabe et al. (58), with one key difference: The Delta spike lacks the N17 glycan sequon due to the T19R mutation (36, 38).

Our WE simulations employ the minimal adaptive binning (MAB) scheme (57), which dynamically identifies bottlenecks and adjusts bin positions during the simulation to more efficiently traverse barriers. As bin positions are tracked throughout simulation, trajectory weights can still be rigorously tracked, however, without adaptive binning, bottleneck regions can be undersampled relative to target end states and when compared to WE protocols conducted with adaptive binning. To ensure comparability between Delta and Omicron RBD opening results performed in this study, we thus also conducted Ancestral RBD opening simulations using this MAB scheme. Moreover, the WE strategy’s primary caveat is the potential to miss slow motions orthogonal to the chosen progress coordinate. For modestly sized systems, it is feasible and advisable to run multiple WE trials with alternative progress coordinates and/or binning schemes to assess the robustness of the observed mechanism. Performing such tests was not practical for systems as large as those considered here. Instead, to demonstrate robustness, we compared results from previously performed Ancestral WE simulations (52) to results from this work, *SI Appendix*, Fig. S1.

Our resulting simulations represent hundreds of μs in aggregate simulation time (79 μs, 34 μs, 36 μs, for Ancestral, Delta, and Omicron spikes, respectively) collected over ~2 mo of continuous running on 64 to 112 A100 GPUs performing hundreds of WE iterations (600, 270, and 400 iterations for Ancestral, Delta, and Omicron spikes, respectively). See *SI Appendix*, Table S2 for complete technical and sampling details. Considering the large computational expense in both simulation time and storage we did not perform explicit replicate simulations, as is routinely done in conventional MD simulations, however it should be noted that each variant WE simulation was launched from an ensemble of 50 coordinates per spike, and each of the hundreds of concurrent walker simulations launched during WE are independent trajectories thus reflecting statistically robust sampling. As a result, we focused on using WE simulations to predict RBD opening pathways, and compare structural differences along said pathways, for Ancestral, Delta, and Omicron spikes.

### Ancestral, Delta, and Omicron Spikes Traverse Distinct RBD Opening Landscapes.

Upon completion of WE simulations for Ancestral, Delta, and Omicron BA.1 spike RBD opening we immediately observed that the three spikes traverse different regions of phase space as defined by the 2D progress coordinates, Fig. 2*B*. During the RBD opening process, as RBD’s open and RBD-core distance increases, the RMSD to 6VSB decreases. All spikes reach a minimum of ~5 Å in RMSD to 6VSB corresponding to an RBD-core distance of ~70 Å in the up state. All RBDs then continue opening beyond 6VSB’s position. As the RBD-core distance increases beyond ~70 Å, the RMSD to 6VSB also increases, reflecting how simulation frames diverge from the 6VSB state as spikes transition into the open and super-open states. To estimate the relative readiness of each spike’s RBD to move out of the closed conformation, we compiled all successful trajectories progressing from closed to up and open target states and calculated the average simulation time required for the transition to occur. These results, Table 1 and *SI Appendix*, Fig. S2, suggest that Delta and Omicron spikes rapidly move into up and open conformations, while Ancestral spikes lag by an average of ~20 to 30 ns when moving to up and open states.

**Table 1.** Mean relative successful pathway sampling times calculated from closed to up and open states, all times listed in ns

Spike	Closed to Up	Closed to Open
Ancestral	40.59 ± 3.17	45.45 ± 1.69
Delta	11.68 ± 2.78	16.53 ± 2.73
Omicron BA.1	17.70 ± 6.54	20.17 ± 4.55

We note these are not kinetic rates but rather qualitative estimates of relative readiness for RBD opening per variant.

Despite overall similarities along the 2D reaction coordinate, distinct differences emerge among variants. The Ancestral and Delta spikes show broader RMSD-to-6VSB variability (5 to 30 Å) across RBD–core distances of 55 to 70 Å, whereas Omicron follows a more direct path with a narrower 5 to 20 Å RMSD range (Fig. 2*B*). All variants sample the up (68 to 72 Å), open (78 to 82 Å), and super-open (>85 Å) states (Fig. 2*C*), but Omicron uniquely advances into the super-open state before reversing, producing conformations with decreasing RBD–core distances. Visualization reveals that the Omicron RBD opens so far that its center of mass moves downward toward the spike’s central plane—like peeling an orange—forming a distinct **“**peel**”** state. Although a full antibody-binding analysis is beyond this work’s scope, this state suggests that Omicron BA.1 may alter antibody interaction patterns, shifting potential contacts from the RBD exterior or RBD–NTD interface to the RBD underside or CH top before ACE2 engagement and S1 release.

### Clustered Opening Pathways Reveal Diversity in Ancestral and Delta Pathways but Similarity in Omicron BA.1 Pathways.

While large ensembles of pathways, as shown in Fig. 2*B*, are invaluable for describing the nuanced variability in RBD opening for Ancestral, Delta, and Omicron spike proteins, these datasets can be cumbersome to analyze (complete trajectories from Ancestral, Delta, and Omicron BA.1, with water molecules and ions removed, are ~660 GB, ~350 GB, and ~320 GB in total size, respectively, *SI Appendix*, Table S1). To mitigate these challenges, we applied the Linguistics Pathway Analysis of Trajectories with Hierarchical Clustering (LPATH) method to cluster all successful RBD opening pathways into distinct routes (*SI Appendix*, section 1.2.2). From these results we identified two pathway routes for each of the Ancestral, Delta, and Omicron spike proteins, Fig. 2*B* (black and gray overlayed trends) and *SI Appendix*, Fig. S3.

As can be seen in Fig. 2*B*, the Ancestral spike’s routes 1 and 2 are very distinct. As the RBD opens (RBD-core distance increases) route 2 first directly minimizes RMSD relative to the 1-RBD-up 6VSB structure, however route 1 initially minimizes RMSD to 6VSB but diverges early on from route 2 at RBD-core distance ~60 Å and begins to increase RMSD to 6VSB before completing the RBD opening. Similarly, the Delta spike’s routes 1 and 2 are distinct in that route 2 follows the lower profile monotonically minimizing RMSD to 6VSB, whereas route 1 diverges from route 2 at RBD-core distance ~70 Å by increasing RMSD to 6VSB. These results suggest Ancestral and Delta spike proteins explore two types of opening pathways: one wherein their RBD position is relatively more similar to 6VSB (route 2 in Ancestral and Delta spikes) and one wherein their RBD positions are distinct from 6VSB (route 1 in Ancestral and Delta spikes). However, the two routes extracted from Omicron RBD opening trajectories are very similar to one another in progress coordinate space: They both minimize RMSD to 6VSB while the RBD is opening. As such, there are likely other conformational changes in Omicron RBD opening which are not described along the 2D progress coordinate space but for which LPATH analysis is sensitive enough to differentiate, i.e., Omicron’s routes 1 and 2 may be distinct along an axis not immediately discernable via the progress coordinate landscape. Interestingly, these results support our hypothesis gleaned from the full landscape results: Omicron RBD opening follows a more direct set of routes with high similarity to one another in early RBD opening stages, whereas Ancestral and Delta RBD opening routes demonstrate lower similarity, higher variability, in early stages of RBD opening. Interestingly, we can also see that routes 1 and 2 for Ancestral and Delta spike proteins both diverge, as described, from one another but that divergence occurs in different regions of progress coordinate space. For the Ancestral spike, route divergence occurs around an RBD-core distance of ~60 Å. For the Delta spike, clear divergence between routes occurs once the RBD has already reached the up state around an RBD-core distance of 70 Å. The implications of Ancestral RBD opening divergence at 60 Å vs. Delta divergence ~70 Å and Omicron’s apparent lack of divergence is not yet clear.

### The N2R and R2N Flexible Linkers Unite All Major S1 Domains.

**Fig. 3.**
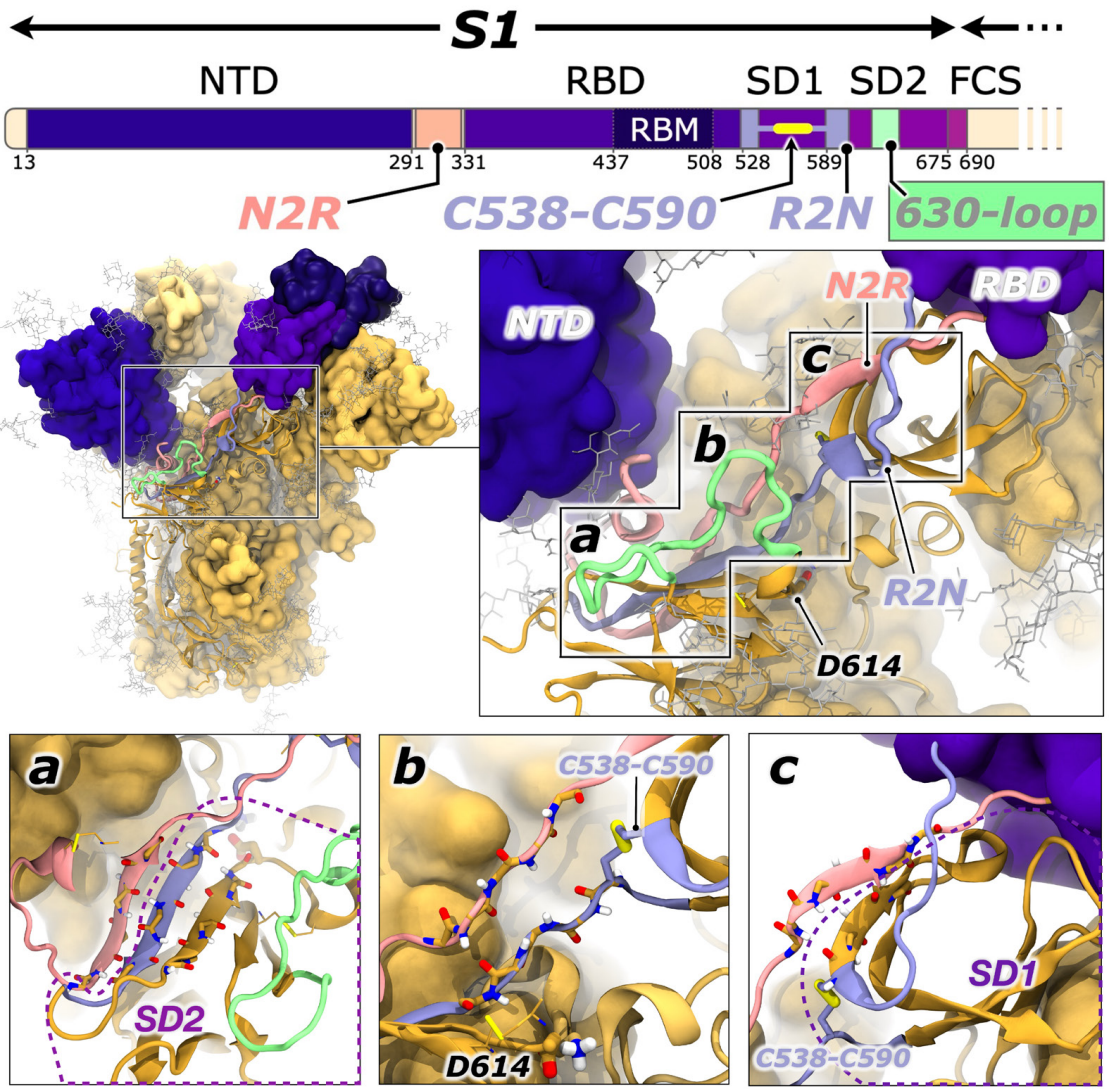
Details of the N2R and R2N linkers and the 630-loop. A one-dimensional sequence of the spike S1 shows how the N2R linker (pink ribbons) connects the NTD directly to the RBD. The R2N linker (lilac ribbons) is revealed when considering the covalent link via the C538 and C590 disulfide bond. The 630-loop (green ribbons) has been described by others to correlate in motion and stability with RBD opening. Panels a, b, and c depict the backbone atoms in licorice (carbon, oxygen, nitrogen, and hydrogen in yellow, red, blue, and white, respectively) to highlight the regions in which the N2R and R2N interact with β-sheets within the SD2 (panel a) and SD1 (panel c) or pass through the 614 proximal region. For clarity, the NTD is not displayed in panel a, and the 630-loop is not displayed in panel b.

Gobeil and Henderson et al. identified conformational correlations between RBD openness and a linker peptide which they designated the NTD-2-RBD (N2R), residues 293 to 330 (59). As can be seen in Fig. 3 (pink ribbon), beginning approximately at position 293, the N2R linker runs from the base of the NTD, serves as a β-strand connected to the SD2 (K310 to T315), forms a short loop (Ancestral) or β-strand sheetlet (Delta and Omicron) (T315 to P322), serves as a β-strand within the SD1 (P322 to N331), and finally passes into the RBD (N331) as a flexible loop around a previously reported transient pocket (60). The RBD itself is then largely well folded into a bundle of α-helices surrounding a central β-sheet and fortified by via several disulfide bonds (C336-C361, C379-C432, C391-C525), including one within the RBM (C480-C488). The one-dimensional spike sequence representation, however, belies the fact that the S1 folds back onto itself: The loop exiting from the RBD (P527-C538, lilac ribbon, Fig. 3) runs adjacent to the SD1 and directly connects to another β-strand (C590 to G594)—the antiparallel β-strand paired with N2R’s T315 to P322 peptide—via a disulfide bond (C538-C590), and then wedges between the base of the NTD and the SD2 via another β-strand (G594 to T602) within the SD2’s central β-sheet. Herein, we will refer to this second linker that connects the RBD back to the base of the NTD via a disulfide bond as the R2N linker (residues 526 to 538, C538-C590, and 590 to 602), which runs parallel to and engages significantly with the N2R (residues 293 to 330). The N2R and R2N both pass over the SD1 to the RBD and ultimately staple to SD2’s β-sheet. In keeping with the trend in previous spike structural biology investigations likening the spike domains to joints—like the ankle, knee, and hip (61)—one could liken the N2R and R2N linkers as “tendons” extending through the spike elbow (NTD to SD2 to SD1) and wrist (SD2 to SD1 to RBD) joints. Gobeil and Henderson et al. show clearly that, for Omicron strain spikes, N2R linker position is highly correlated with RBD openness (59).

Gobeil et al. (59) and Manrique et al. (62) have also identified loops within the N2R as likely important in facilitating spike RBD conformational changes. Furthermore, despite the fact its position is far from the RBM (~75 Å), several groups have reported the D614G mutation correlated significantly to increased infectivity of SARS-CoV-2 (23–33, 63). Therefore, the relationship between position 614, ACE2 binding, and SARS-CoV-2 infectivity suggests long-range allosteric communication. While not located in the N2R or R2N, the 614 position is directly proximal to a β-sheetlet within the N2R and R2N (T315 to P322 and C590 to G594, respectively). Thus, considering the likelihood of N2R and R2N linkers facilitating allostery within the S1, and the proximity of these linkers to the 614 position, we then sought to characterize correlated motions and interaction differences within these linkers. Furthermore, Zhang et al. (28) and Dokainish and Sugita (33) have noted distinct structural and dynamical changes for the so-called 630-loop within the SD2: This loop extends off the β-turn where 614 sits, moves around the SD2 and reconnects to the β-sheet. Dokainish and Sugita (33) showed that not only was the position of the loop correlated to RBD opening but also that the D614G mutation, or protonation of D614, altered stability of the 630-loop allowing tighter packing toward the spike core (33). Zhang et al. also showed the 630-loop becomes more ordered in Omicron and later spikes (64).

### Enhanced Allosteric Communication within Delta and Omicron Spikes.

**Fig. 4.**
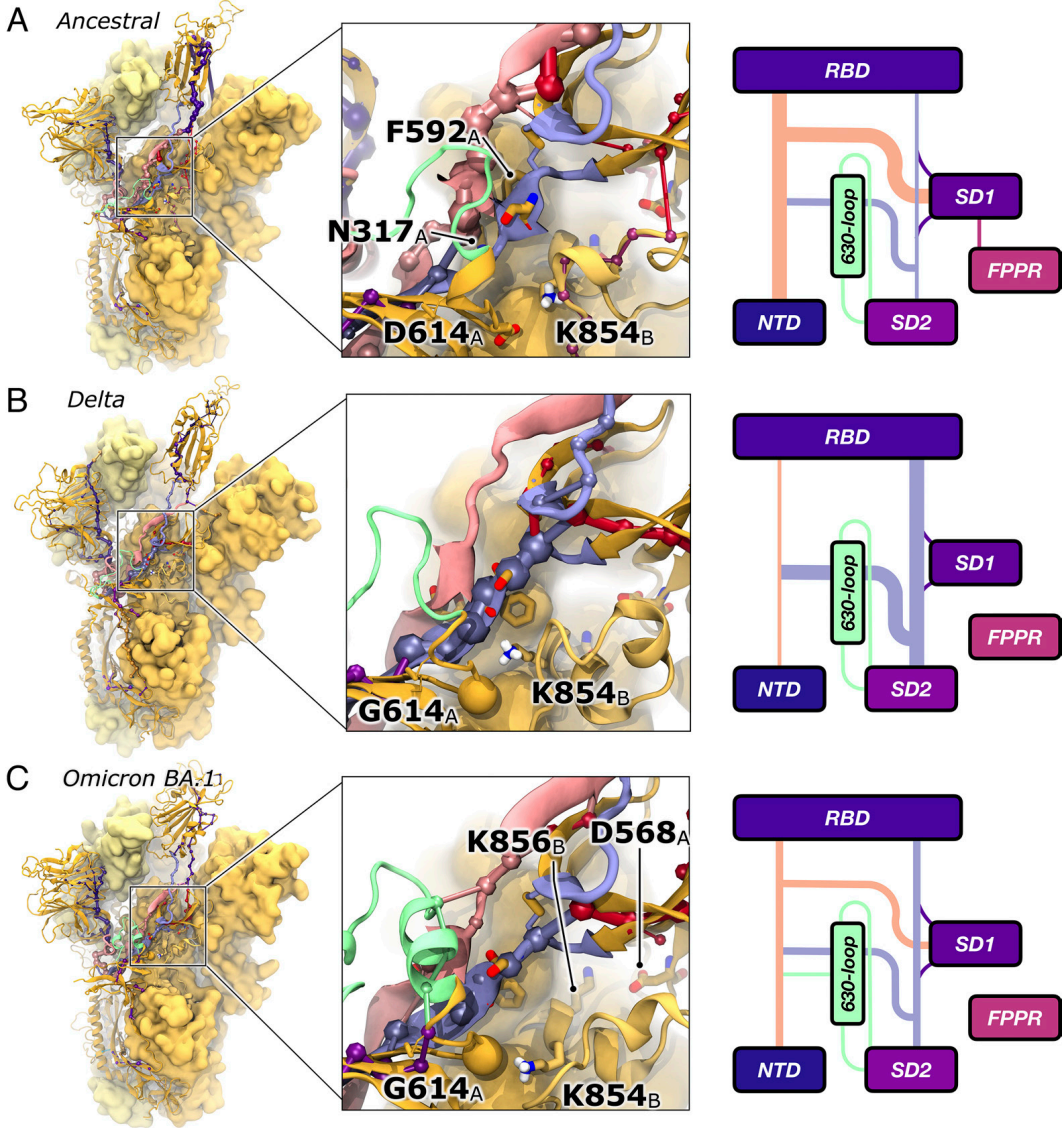
Dynamical networks reveal a lane of communication in Delta and Omicron spike proteins. Dynamical networks calculated for (*A*) Ancestral, (*B*) Delta, (*C*) Omicron BA.1 spike proteins from the most probable opening pathways. In all panels, the spike is represented in yellow ribbons (chain A) and medium- and light-yellow surfaces (chain B and C, respectively). Chain B’s FP and FPPR are shown in yellow ribbon to reveal positions of K854. Networks are shown as spheres and edges and colored according to which domain or linker the node belongs, for Ancestral, Delta, and Omicron spikes, respectively. Amino acids of interest (residues 614, 317, 592, and 854) are represented in licorice with yellow carbon atoms. Schematics detail simplified representations of correlated motions between domains via the N2R, R2N, and 630-loop. For full detail, see *SI Appendix*, Schematics S2–S4.

To identify and evaluate the strength of allosteric communication networks between linkers and domains during RBD opening, we performed dynamical network analyses using the Weighted Implementation of Suboptimal Paths (WISP) (65). Using graph theory, we constructed residue-based networks using cross-correlation analysis where nodes and edges are weighted by their strength of correlated motions (65) (for complete methodological description see *SI Appendix*, section 1.2.5). This approach therefore not only predicts allosteric networks but also estimates how the strength of correlation within these networks can change as a function of the sequence mutations and structural rearrangements as seen in variant spikes. For all spike RBD opening routes, we see differences in the predicted allosteric networks within the chain undergoing RBD opening as a function of variant, Fig. 4, as well as differences within the nonopening chains and between chains, *SI Appendix*, Fig. S3. Herein, we will describe differences seen within the RBD-opening chains for the routes with highest probability (as taken by the relative weight per route trajectory selected via LPATH). As shown in Fig. 4*B*, for the Delta spikes, allosteric communication is relayed between the NTD and RBD via the R2N linker (lilac spheres and rods), and for the Omicron (Fig. 4*C*) spike allosteric communication is relayed via the N2R (pink spheres and rods) and the R2N linkers. However, for the Ancestral spike, the allosteric pathway connecting the NTD to the RBD relies primarily on the N2R (Fig. 4*A*), with very little communication through the R2N. Interestingly, in the Ancestral spike, only one node within the R2N is activated in the less probable route 2 (*SI Appendix*, Fig. S4*A*) where a strong degree of correlated motion is observed between residues N317 and F592.

Conversely, in Delta spike’s most probable RBD opening route (2), the R2N facilitates the allosteric communication between the NTD and RBD, Fig. 4*B*, with very little contribution from the N2R. Delta’s less probable, but still likely, route 1 does reveal significant communication within the N2R, *SI Appendix*, Fig. S4*B*. Thus, the precise RBD to NTD communication network may vary depending on the pathway traversed by the RBD during opening, but for Delta spikes the R2N is the primary facilitator of this allosteric information. Interestingly, in both of Omicron’s RBD opening routes, we observe clear and robust communication through both N2R and R2N linkers, Fig. 4*C*. In fact, communication networks for both Omicron routes show only very minor differences. This is consistent with earlier descriptions of the complete conformational landscapes collected from Omicron simulations, wherein the Omicron RBD opening follows a “tighter” probability profile, transitioning more directly from closed to partially up and open before exploring broader conformational space in the super-open and peel states (Fig. 2*A*). Omicron’s nearly identical allosteric networks for both pathway classes suggests that early stages of Omicron RBD opening follow a more consistent route compared to Ancestral and Delta, where the RBD explores a broader range of positions relative to the 6VSB structure (RMSD to 6VSB).

Contrary to Delta and Omicron, allosteric networks in the Ancestral spike rely largely on correlation through the N2R only: 34.3% and 38.4% of total weights for nodes within Delta and Omicron allosteric networks pass through the R2N, respectively, compared to 11.4% through the Ancestral spike’s R2N. Additionally, Delta and Omicron spikes exhibit more balanced communication between N2R and R2N linkers compared to the Ancestral spike: Node weights for the N2R and R2N within Ancestral spike were 87.8% vs. 11.4%, compared to 64.8% and 34.3% for Delta, and 58.7% and 38.4% for Omicron. Thus, much like opening new lanes on a highway to alleviate congestion, significant degrees of communication flows through both the R2N in Delta and Omicron spikes during RBD opening, likely allowing for more direct, concerted, and attenuated communication pathways between NTDs and RBDs. Additionally, the Omicron spike’s 630-loop is directly connected to the RBD-to-NTD allosteric network, whereas this same region is largely disconnected from this allosteric network in Ancestral and Delta RBD opening pathways, Fig. 4 and *SI Appendix*, Figs. S4 and S5. As mentioned, several groups have posited a correlation between the 630-loop motion and stability to RBD opening (28, 33). Here, we see that Omicron’s 630-loop is very well ordered with two additional α-helical turns relative to the Ancestral spike. Additionally, we see that during RBD opening, the 630-loop stays far more compacted toward the spike core in Omicron spikes than in Delta or Ancestral spikes and that compaction toward the core only increases (decreasing distance from core) during RBD opening on the chain that is opening, *SI Appendix*, Fig. S6.

### The D614 to K854 Salt Bridge Dampens S1 Allostery and Must Break before the Ancestral Spike’s RBD Can Open.

Communication along the R2N in the Ancestral spike is likely hindered due to steric congestion caused by a neighboring salt bridging interaction between D614 and K854. As mentioned previously, the N2R and R2N linkers pass directly “behind” the 614 position. We sought to characterize the degree to which this salt bridge and the 614 position may impact allostery through the 614 proximal region. Cryo-EM resolution of the D614G spike structure, and comparisons of this structure to Ancestral strain spike structures, reveal potential interactions between D614 and two residues, T859 and K854, within the neighboring chain’s FPPR (29). These interactions have been posited as likely stabilizing the RBD while it is opening (28). Considering the potential of the D614G mutation to alter spike allostery, we carefully probed the region around the 614 position to uncover any relationship to RBD dynamics.

**Fig. 5.**
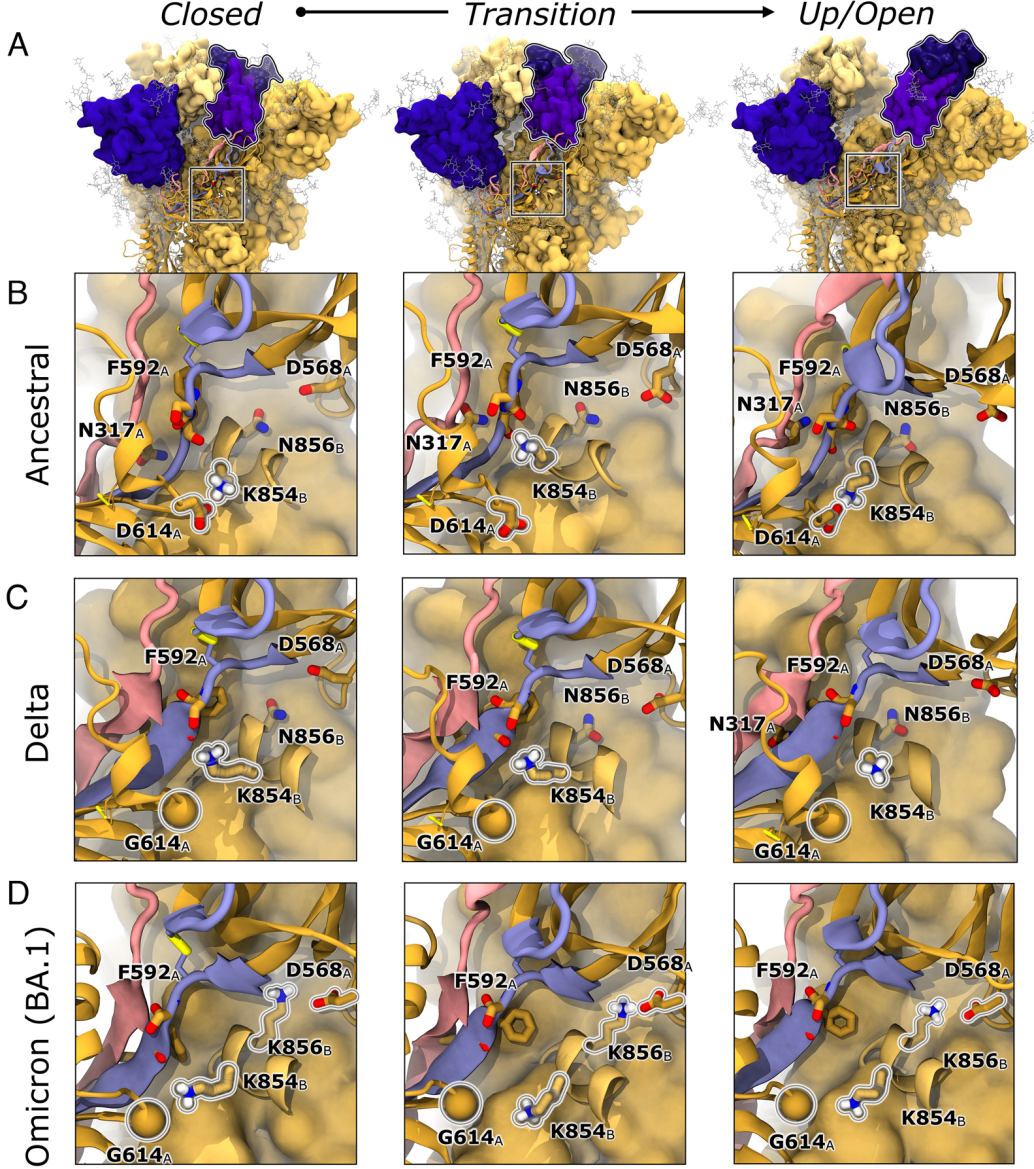
RBD opening progress as a function of salt bridging interaction between D614 and K854. In all panels, the spike is represented in dark yellow ribbons (chain A) and medium- and light-yellow surfaces (chain B and C, respectively). The FPPR of chain B is represented as a medium yellow ribbon to reveal positions of K854 and N/K856. The N2R (residues 293 to 330) and R2N (residues 526 to 538, C538-C590, and 590 to 6023) are highlighted in pink and lilac ribbons respectively. Amino acids of interest (residues 614, 317, 592, 854, 856, and 568) are represented in licorice with dark and light yellow carbon atoms indicating chain A or B, respectively. Disulfide bonds are highlighted with thick yellow licorice. The G614 position in Delta and Omicron spikes is denoted with a yellow sphere. (*A*) Frames from Ancestral spike opening trajectories were selected from closed, transition, and open states to highlight RBD opening progress correlating with the zoom-in images on the 614 proximal region for the (*B*) Ancestral, (*C*) Delta, and (*D*) Omicron BA.1 spike proteins. In the closed and open states for the Ancestral spike, the D614 to K854 salt bridge is tightly formed. Before the RBD can begin to open the D614-K854 salt bridge breaks and remains broken until the RBD enters the up conformation (*SI Appendix*, Figs. S7 and S15*B*). For Delta and Omicron spikes, the D614 to K854 salt bridge is ablated and thus there is no reliance on that interaction breaking before RBD opening. In the Omicron spike, a new salt bridge is established between K856 and D568, and this salt bridge remains intact throughout RBD opening.

Upon first observing our successful RBD opening simulations, we quickly identified that in the closed Ancestral spike, D614 engages the neighboring chain’s K854 in a tight salt bridge, Fig. 5 *A* and *B*. As can be seen in *SI Appendix*, Figs. S7 and S15*B*, for the Ancestral spike, the D614-K854 salt bridge is tight in the closed state (RBD-core distance < 55 Å) but breaks in initial stages of transition from closed to up states (RBD-core distances 55 to 65 Å). The D614-K854 salt bridge then reforms in later stages of this transition (RBD-core distances 65 to 68 Å) and is again tightly engaged when the RBD is in the up state (RBD-core distances 68 to 72 Å). Consistent with our results, several Cryo-EM spike structures in up and open conformations show the D614-K854 salt bridge is either tightly engaged (28, 66), or could be engaged if the FPPR were resolved, but our simulation results can capture transition state conformations wherein the salt bridge breaks and reforms in the 1up RBD state. Beyond the Ancestral spike’s up state, as a function of the 2D conformational landscape, the D614-K854 salt bridge occupies regions of tight, light, or no engagement depending on RBD position. Furthermore, in all successful pathway trajectories for Ancestral spike opening, we noted that the D614-K854 salt bridge breaks before the RBD can make significant progress along the opening pathway. As described earlier, the Ancestral spike spends significant amounts of time (44.5 ± 3.1% of frames, *SI Appendix*, Fig. S15*A*) per successful opening trajectory in the closed conformation (RBD-core distance < 55 Å). Most of the Ancestral spike’s closed frames show a tightly engaged salt bridge (93.1 ± 0.0% of all closed frames from successful opening trajectories have D614-K854 salt bridge distance < 3 Å), *SI Appendix*, Fig. S7. In fact, most Ancestral opening pathways spend ~17 ns of molecular time prior to finding the first broken D614-K854 salt bridge distance (D614-K854 distance > 4 Å), and only another ~5 ns of molecular time (total ~22 ns) before the salt bridge sustains a broken conformation (more frequent than every 4th frame with a D614-K854 distance > 4 Å). At ~24.5 ns, with the D614-K854 salt bridge in the sustained-broken conformation, the Ancestral spike moves out of the closed state into the transitional state heading toward the up state (RBD-core distance > 55 Å, Fig. 5*B* and *SI Appendix*, Figs. S7 and S15 *A* and *B*). Around 30 ns of molecular time, D614 and K854 move even further apart (>5 Å) and concomitantly the Ancestral RBD progresses linearly (~0.8 Å/ns) toward the up state (RBD-core distances 55 to 62 Å, *SI Appendix*, Figs. S7 and S15 *A* and *B*). At ~36 ns, the D614-K854 salt bridge reforms (<4 Å), slowing down but not halting RBD opening at ~0.5 Å/ns (RBD-core distances 62 to 68 Å, Fig. 5*B* Up/Open and *SI Appendix*, Figs. S7 and S15 *A* and *B*). As described above, in the RBD up state (RBD-core distances 68 to 72 Å, *SI Appendix*, Fig. S7) the D614-K854 salt bridge reforms, again as seen in several Cryo-EM structures (28, 66). Beyond the up state, there are several pockets of conformational space in which the D614-K854 salt bridge is more or less engaged, suggesting RBD position in the Ancestral spike is highly correlated with the strength of this interaction.

Conversely, the D614G mutation, as seen in Delta and Omicron spikes, ablates the salt bridge to K854. As such, for Delta and Omicron RBD opening simulations we see no correlation between G614-K854 distance and RBD-core distance or molecular opening times, Fig. 5 *C* and *D* and *SI Appendix*, Figs. S7 and S15 *A* and *B*. Additionally, as mentioned, Delta and Omicron spikes spend very little time in the closed state, (11.4 ± 2.9%, 17.3 ± 5.2% of RBD opening frames with <55 Å RBD-core distance, respectively) and instead begin to open quickly at ~1.3 Å/ns and ~1.2 Å/ns respectively (*SI Appendix*, Figs. S7 and S15 *A* and *B*).

We also probed neighboring interactions around 614’s position, particularly any interactions with the N2R and R2N linkers. We saw that the Ancestral spike’s D614-K854 salt bridge breaking event was concomitant with a backbone twisting (as identified from φ/ψ distributions) observed for R2N-linker residues P589 to G594 (PCSFGG, *SI Appendix*, Figs. S8 and S15 *E*–*H*), particularly S591 and F592, which is not observed for Delta or Omicron RBD opening pathways. Furthermore, in the Ancestral spike, we see an increase in contacts between N2R-linker residue N317’s side chain and R2N-linker residue F592’s backbone after the φ/ψ switch (*SI Appendix*, Figs. S9 and S15*D*) where this contact is not seen in Delta and Omicron spike proteins. Interestingly, during the RBD transition into the up-state, after the D614-K854 salt bridge reforms, backbone φ/ψ distributions do not revert to their conformation in the closed state, *SI Appendix*, Figs. S8 and S15 *E*–*H*. Considering this backbone twist was only seen for Ancestral spikes, and its correlation with D614-K854 salt bridge breaking, we hypothesized that local steric congestion could be impacting R2N dynamics. Thus, we calculated the distance between the SD2 and R2N (particularly residues 590 to 594, *SI Appendix*, Fig. S11), the R2N (residues 590 to 594) and the neighboring chain’s FPPR (*SI Appendix*, Fig. S12), the SD2 and the neighboring chain’s FPPR (*SI Appendix*, Fig. S13), and the area between these three domains (*SI Appendix*, Fig. S14). For the Ancestral spike, we see that R2N residues 590 to 594 are closer to the neighboring chain’s FPPR by ~1 to 4 Å than in Delta and Omicron spikes in the opening protomer. We also see that the area between the SD2, R2N, and neighboring FPPR is ~10 to 20 Å^2^ closer than that seen in Delta and Omicron spikes. Moreover, the area between the SD2, R2N, and the FPPR increases during RBD opening in a profile similar to that seen for the breaking of the D614-K854 salt bridge, *SI Appendix*, Fig. S15 *J*–*L*. Moreover, our contact and network analysis has elucidated residues (D614, D568, S591-F592) at or near positions identified in other previous work (D614, A570, and G601) wherein Ancestral spike RBD opening pathways were predicted via steered MD and umbrella sampling (67). Taken together, these results suggest the D614-K854 salt bridge induces local steric congestion within the D614 proximal region around, and likely impeding optimal allosteric communication within, the R2N linker.

Due to our observation that the D614-K854 salt bridge impacts not only local contacts but linker backbone conformations and general degree of subdomain compactness, we sought to characterize the relative flexibility between variant spikes (Delta/Omicron) and the Ancestral spike along their RBD opening routes 1 and 2. Using the Maximum Information Spanning Tree (MIST) approach implemented in PDB2ENTROPY package (*SI Appendix*, section 1.2.8) (68), we calculated relative flexibility for all residues within each spike along the entirety of clustered successful routes 1 and 2 trajectories and calculated percent differences in flexibility per residue between Delta and Ancestral routes 1 and 2 (*SI Appendix*, Eq. **S1**). To avoid artificially inflating flexibility differences due to Ancestral route 1 occupying an alternative region of conformational space not observed by either Omicron BA.1 route 1 or 2, both Omicron routes were compared to Ancestral route 2, as these routes all occupy similar 2D progress coordinate space to one another. A complete discussion and description of these results can be found in *SI Appendix*, section 2.3.0. Briefly stated here: Most residues within 20 Å of the D614-K854 salt bridge are more flexible during RBD opening in variant spikes than in Ancestral spikes (94.9%, 71.8%, 59.1%, and 60.8%, for Delta route 1, Delta route 2, Omicron route 1, and Omicron route 2, respectively). The increased flexibility in this region for variant spikes is directly related to the alleviation of congestion of 614 proximal region caused by the tight D614-K854 salt bridge present only in the Ancestral spike.

Considering this relationship between the D614-K854 salt bridge contact profile during RBD opening (*SI Appendix*, Figs. S7 and S15*B*), R2N’s observed backbone twist particularly at positions S591 and F592 (*SI Appendix*, Figs. S8 and S15 *E*–*H*), significant contacts between N2R-linker residue 317 and R2N-linker residue 592 (*SI Appendix*, Figs. S9 and S15*D*), the decreased SD2-FPPR-R2N area for Ancestral spikes relative to Delta and Omicron (*SI Appendix*, Figs. S11–S14), and the increased flexibility for residues around the D614-K854 salt bridge for variant spikes relative to the Ancestral strain (*SI Appendix*, Table S3 and Figs. S16 and S17), the strong D614-K854 salt bridge in the Ancestral spikes dampens dynamics in this region and disrupts potential R2N facilitated allosteric communication, instead requiring all communication to pass only through the N2R-linker. Breaking of this salt bridge via the D614G mutation, alleviates frustration in the 614-proximal region, and opens communication along the R2N as seen for Delta and Omicron spikes. In addition, as described above, the Ancestral RBD does not begin opening until the D614-K854 salt bridge breaks. As a result, when considering all successful opening pathways, the Ancestral spike spends ~44.5 ± 3.1% of frames in the closed state and 92.7 ± 0.0% of that time is spent iterating until a sustained broken salt bridge conformation (>4 Å, more frequent than every 4th frame) is found allowing the RBD to begin to emerge from the down state (RBD-core distance > 55 Å). Conversely, Delta and Omicron spikes spend only 11.4 ± 2.9% and 17.3 + 5.2% of successful opening frames, respectively, in the closed state before their RBDs begin to emerge and we see no relationship between G614-K854 distance and RBD opening, Fig. 5 and *SI Appendix*, Fig. S7. Thus, ablation of the salt bridge by D614G opens a lane of allosteric communication along the R2N in addition to the N2R, potentially contributing to faster RBD opening for D614G spike variants via concerted correlated motions through both linkers (*SI Appendix*, Fig. S5), consistent with our relative molecular opening times (Table 1 and *SI Appendix*, Fig. S15*A*). Our results are consistent with those from Manrique et al. wherein they performed conventional MD simulations of spikes in the closed state. Their results reveal several residues within the N2R have high “betweenness” along allosteric communication networks, indicating their importance (62). Our work builds on their results, as we have confirmed the N2R allosteric network, even in the presence of N- and O-linked glycans, across several spike variants, and support that such communication networks are vital for RBD opening. Additionally, several groups have also shown the D614G mutation results in an increase in flexibility in the surrounding region, likely leading to increased RBD flexibility (24–26, 28, 29, 31–33). Taken together, these results strongly suggest that D614G increases RBD opening rates by alleviating conformational frustration around the R2N in the 614 proximal region, thereby affording concerted and consistent NTD to RBD allostery via both N2R and R2N linkers.

Additionally, upon visualizing Omicron RBD opening trajectories we also observed the establishment of a new salt bridge between FPPR residue K856 (result of N856K mutation) and the neighboring chain’s D568, Fig. 5*D*. We calculated the strength of this salt bridge during Omicron RBD opening and see that it is tightly engaged throughout all regions of the 2D progress coordinate space, *SI Appendix*, Figs. S10 and S15*I*. Due to the presence of the bulky, polar, yet uncharged N residue at the 856 position, Ancestral and Delta spikes demonstrate short N856-D568 distances, but those short distances do not reflect tight engagement and are not correlated to RBD-core distance. The 856 position is within the FPPR and near K854. Our results suggest the K856-D568 salt bridge may allow for restabilization of the FPPR via reintroduction of the salt bridge while still affording necessary flexibility around, and allosteric communication through, both N2R and R2N linkers.

### HDXMS Confirms D614G Altered Dynamics Around Spike Allosteric Networks.

**Fig. 6.**
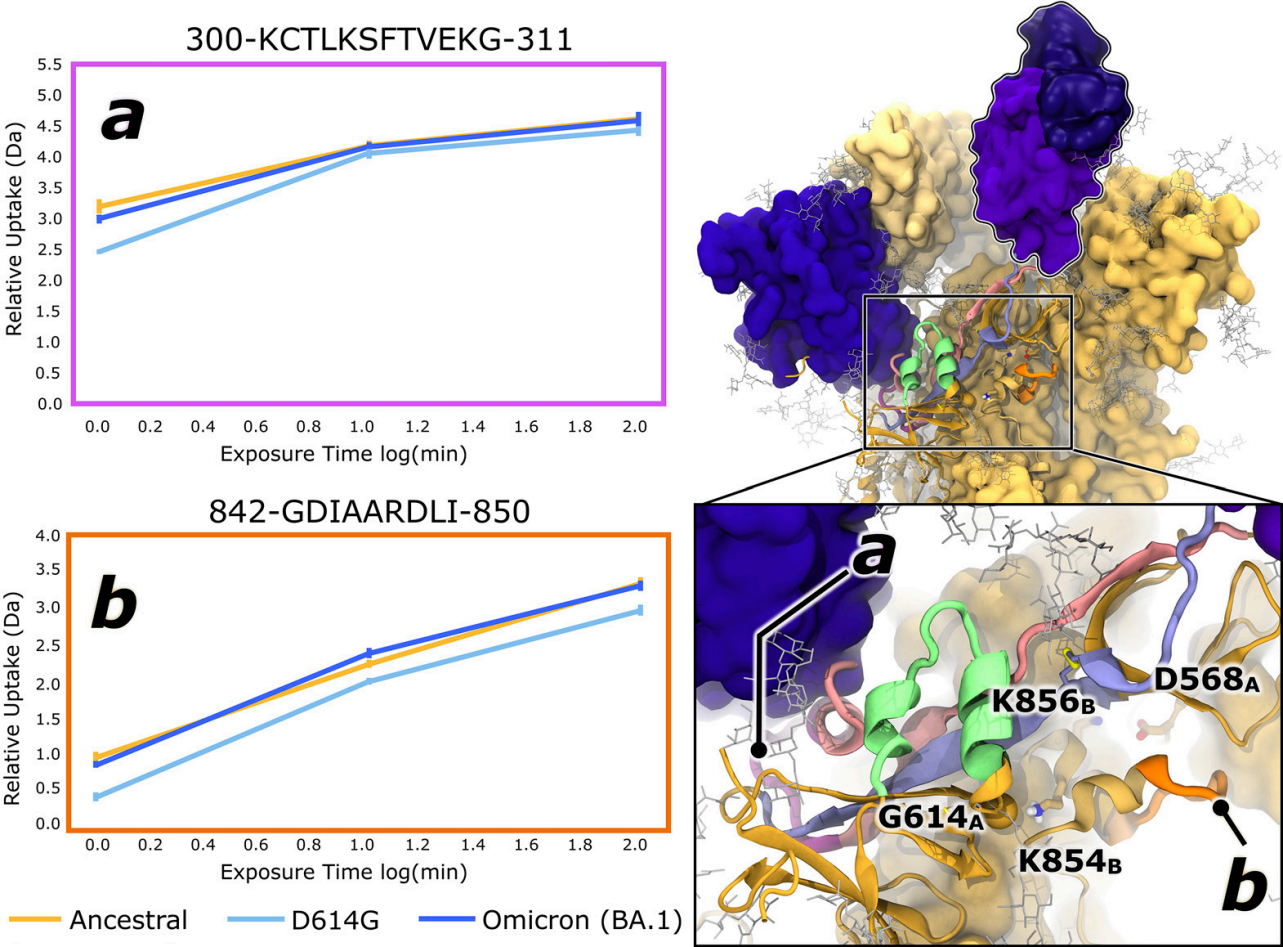
HDXMS reveals structural and dynamic changes near the 614 region. The Omicron BA.1 spike structure (*Right*) shows peptides A and B relative to the NTD, RBD, FPPR, N2R, and R2N. Chain A is displayed as yellow ribbons; chains B and C as medium- and light-yellow surfaces. As in Figs. 2 and 3, the N2R, R2N, and 603 loops are highlighted in pink, lilac, and green, respectively. Key residues (D568, D614, K854, N856) are shown in licorice, with dark or medium-yellow carbons for chains A and B; disulfides are depicted as thick yellow licorice. Peptides A and B are marked in magenta and orange ribbons. Relative deuterium uptake plots (*Left*) show HDXMS data for peptides A and B (in Daltons vs. log[min]) comparing Ancestral (yellow), D614G (cyan), and Omicron BA.1 (blue) spikes.

Considering the contact and communication changes observed in N2R and R2N linkers around position 614, we sought to experimentally probe dynamics in this region via hydrogen/deuterium exchange mass spectrometry (HDXMS) from SARS-CoV-2 spikes embedded on virus-like particles (VLPs) for Ancestral, D614G, and Omicron BA.1 strain spikes. (Note: Each spike represents the circulating genetic sequence for the given variant of concern, i.e., recombinant spikes incorporating proline substitutions were not used, see *SI Appendix*, Table S4 and section 1.3). It should also be noted that we maintained glycans on spike samples during HDXMS experiments, i.e., we are not employing a PNGase column to cleave glycans from the backbone. However, the presence of glycans decreases peptide coverage due to differential uptake on glycosylated residues. Overall, we obtained 53 pepsin peptide fragments spanning 44.9% of the spike sequence as described in Gramm et al. (69) Within this dataset, we identified two peptides near the D614G proximal region and the N2R/R2N linkers which were not discussed in Gramm et al. (69) We identified coverage of two peptides near D614G and N2R/R2N linkers. Peptide A (Fig. 6*A*), residues 300 to 311, contains a short α-helix leading to a flexible loop located at the base of the NTD. Peptide B (Fig. 6*B*), residues 842 to 850, is within the fusion peptide proximal region (FPPR, residues 834 to 855), is near to position 854, and includes a loop and an α-helix. While HDXMS is limited by peptide level resolution, uptake values for representative peptides provide an average readout of dynamics that can be correlated to changes in flexibility observed in or simulations for these regions (70). Ancestral and Omicron spikes exhibit a similar degree of deuterium uptake for Peptides A and B, while D614G spikes on SARS-CoV-2 VLPs show dramatically decreased deuterium uptake, especially for Peptide B and at early time points.

Our HDXMS results support that the D614G mutation impacts NTD and FPPR dynamics on the minutes timescale, which is consistent with the experimentally observed rate for RBD opening, which is on the order of seconds (71). Considering our simulation results demonstrating the loss of the salt bridge contact and enhancement of communication along the R2N, the base of the NTD and the FPPR loop compensate via rigidification, thus explaining loss of deuterium uptake in these regions in D614G compared to Ancestral spike proteins. However, with reestablishment of a salt bridge in the region with Omicron’s N856K mutation, Peptides A and B can return to similar degrees of flexibility as seen in the Ancestral strain. Although a comprehensive characterization of backbone hydrogen bonding in this region is outside the scope of this current work, our HDXMS results confirm that D614G and K856N mutations are key to explaining changes in spike flexibility within this region and, in context with simulation results, the increased number of RBDs in the up state in later VOCs relative to the Ancestral strain.

## Conclusions

We used WE simulations to map RBD opening pathways in the Ancestral (2019), Delta, and Omicron BA.1 spike glycoproteins. In the Ancestral spike, RBD opening requires breaking the D614-K854 salt bridge, which reforms in the up, open, and super-open states. The D614G mutation abolishes this salt bridge, eliminating the dependence of RBD opening on its disruption. We also identified an allosteric communication network linking the NTD and RBD—consistent with Manrique et al. (62)—passing through linkers near position 614. Substitution to glycine at this site establishes a communication network, suggesting that the D614-K854 salt bridge constrains optimal S1 allostery during RBD opening.

In Omicron BA.1, the N856K mutation within the FPPR likely restores a nearby salt bridge to D568 complementing D614G. Together, these mutations appear to fine-tune flexibility and stability across the allosteric network. We also observed a previously unseen “peel” conformation unique to Omicron BA.1, in which the RBD flexibly separates from the S2 core—potentially priming faster membrane fusion and altering antibody. Finally, Figs. 1–6 highlight multiple N-linked glycans across the NTD (at positions N17, N61, N74, N122, N149, N165, N234, N282), RBD (N331, N343), and SD2 (N603, N616), along with two potential O-linked sites on the N2R linker (T323 and S325). Newby et al. (72) recently reported glycoprofile shifts among Ancestral, Delta, and Omicron BA.1 spikes, including loss of N17 in Delta and the changes in N-glycoforms throughout.

Together, these results reveal two linkers, N2R and R2N, that mediate allosteric communication within the spike’s S1 domain. In the Ancestral spike, the D614-K854 salt bridge causes steric congestion near position 614, restricting communication to the N2R linker. The D614G mutation relieves this frustration, enabling coordinated signaling through both linkers. We predict that Delta and Omicron BA.1 spikes, each containing D614G, exhibit faster RBD opening and greater propensity for 1-up, 2-up, and 3-up conformations, consistent with enhanced ACE2 binding.

These findings clarify the long-debated role of the D614-K854 salt bridge in reshaping the RBD conformational landscape. By tracing how such mutations confer thermodynamic and kinetic advantages, we gain insight into viral fitness and the emergence of function-enhancing mutations in spike and other viral glycoproteins. Structural comparisons among variants also aid in predicting epitope accessibility and immune evasion. Overall, our results define how D614G remodels spike structure, dynamics, and function.

## Methods

### Simulations and Analysis Methods.

For a detailed description of spike model construction and complete details of all analysis methods please refer to *SI Appendix*, sections 1.0–1.2. All WE simulations were performed with the WESTPA (73) software and GPU-accelerated Amber dynamics engine (74–77), using the CHARMM36m all-atom force field (78–80) and CHARMM-modified TIP3P water model.

HDXMS Methods: For complete details of HDXMS methods, including VLP preparation, please see previous work by Plescia et al. (81) and *SI Appendix*, section 1.3.0.

## Supplementary Material

Appendix 01 (PDF)

## Data Availability

This work used standard builds of the WESTPA 2020.02 software (https://github.com/westpa/westpa) (82) and Amber 18 (https://ambermd.org) (83). Simulations were performed according to best practices for running WE simulations (84). MAB binning was used according to Torrillo et al. (57). A tar ball containing selected WE simulation trajectories (psf/pdb/dcd), along with input scripts (Amber and NAMD) and MDAnalysis scripts (py), is available for download on the Amaro Lab website (https://amarolab.ucsd.edu/data.php#covid19) (85) with the following name: D614G_for_sharing_amarolab.tar.gz. Some study data are available upon request.
